# Hyaluronic acid regulates cellular UDP-GlcNAc levels through CD44 to affect glycosylation and cell biological functions

**DOI:** 10.1016/j.jbc.2025.111111

**Published:** 2025-12-27

**Authors:** Yue Wang, Tomoya Isaji, Tiangui Wu, Takuro Ono, Tsukushi Saito, Yoshiyuki Kuroda, Tomohiko Fukuda, Jianguo Gu

**Affiliations:** 1 Division of Regulatory Glycobiology, Graduate School of Pharmaceutical Sciences, Tohoku Medical and Pharmaceutical University, Sendai, Miyagi, Japan; 2 Institute of Molecular Biomembrane and Glycobiology, Tohoku Medical and Pharmaceutical University, Sendai, Miyagi, Japan; 3 School of Nursing & Health, Aichi Prefectural University, Nagoya, Aichi, Japan

**Keywords:** CD44, hyaluronic acid, *N*-glycan, *O*-GlcNAcylation, UDP-GlcNAc

## Abstract

Hyaluronic acid (HA) is a key component of the extracellular matrix. Higher HA levels are strongly associated with poor prognosis in advanced cancer. Notably, the biosynthesis of *N*-glycans, *O*-GlcNAc, and HA all depend on UDP-GlcNAc as the essential donor substrate. Therefore, there may be functional relationships among different glycan types, although the specific mechanisms behind these interactions remain unclear. We established knockout (KO) cell lines for hyaluronan synthase 2 (HAS2) and CD44 in HeLa and PANC-1 cell lines, which express relatively high levels of HAS2. Results from cell proliferation, Transwell, wound-healing, and colony assays showed that proliferation, migration, and clonogenic capacity were significantly reduced in HAS2- or CD44-KO cells compared to wild-type cells. Lectin blot and HPLC analyses revealed increased levels of intracellular UDP-GlcNAc, *O*-GlcNAcylation, and GlcNAc-branched *N*-glycans in HAS2 KO cells. These changes were reversed by adding exogenous HA to HAS2 KO cells or by restoring HAS2 expression. Interestingly, HA effects were not observed in CD44 KO cells, indicating the key role of CD44 in mediating these HA-induced changes. Additionally, CD44 KO significantly reduced β-catenin levels and cell migration, which could be rescued with a β-catenin activator. Our findings suggest that cells sense extracellular HA levels through CD44 to induce CD44-dependent β-catenin signaling, potentially regulating fructose-6-phosphate amidotransferase, a rate-limiting enzyme in the hexosamine biosynthetic pathway responsible for the synthesis of UDP-GlcNAc. These results provide a potential mechanistic connection between extracellular HA and intracellular glycosylation, offering new insights into the diverse roles of HA in cell biology.

Hyaluronic acid (HA) is a linear glycosaminoglycan and a major component of the extracellular matrix (ECM) (1, 2). HA has a high water-retention capacity, which contributes to tissue elasticity and lubrication (3). The level of HA accumulation is strongly associated with a poor prognosis in patients with advanced cancer (4, 5, 6). For example, in prostate cancer stroma, increased HA levels promote tumor proliferation and are linked to poor outcomes (7). Clinical studies have reported increased levels of low-molecular-weight HA in highly invasive breast cancer (8). Additionally, high HA concentrations have been detected in the urine of patients with high-grade bladder cancer (9), and in patients with squamous cell carcinoma, a positive correlation was revealed between CD44 expression and HAS levels (10). CD44 is a well-characterized HA receptor and a diagnostic biomarker (11, 12, 13, 14).

In mammalian cells, the genome contains three HAS genes, which encode three distinct isoenzymes named HAS1, HAS2, and HAS3 (15). The specific roles of each HAS isoenzyme in various biological processes are still being studied. However, HAS2-null mice are non-viable due to severe cardiac abnormalities, and the catalytic properties of HAS2 enable highly efficient HA synthesis (16, 17). Therefore, HAS2 is widely regarded as the most critical HA synthase (18). HAS synthesizes HA at the plasma membrane using uridine diphosphate glucuronic acid (UDP-GlcA) and UDP-*N*-acetylglucosamine (UDP-GlcNAc) as substrates (15).

Protein glycosylation is one of the most common and complex post-translational modifications (PTMs) in eukaryotic cells (19, 20); its dynamic regulation is especially important in the context of cancer pathophysiology. The main types of protein glycosylation are *N*-linked and *O*-linked glycosylation, as well as *O*-GlcNAcylation. *N*-linked glycans are covalently attached to asparagine (Asn) residues of membrane-bound or secretory proteins (21). The formation of complex branched structures in the Golgi apparatus is mediated by a family of *N*-acetylglucosaminyltransferases (GnTs), which utilize UDP-GlcNAc as the glycosyl donor (22). Alterations in glycosylation play a critical role in tumor progression and are considered a hallmark of cancer (23). Notably, overexpression of GnT-V (MGAT5) enhances the GlcNAc-branched *N*-glycans of *N*-cadherin, promoting phosphorylation of β-catenin *via* the EGFR and Src signaling pathways (24). This cascade weakens cell–cell adhesion and promotes increased cellular motility (25). Furthermore, upregulation of GnT-V has been detected during the early stages of hepatocellular carcinoma, suggesting its involvement in tumorigenesis (26). Similarly, aberrant overexpression of GnT-IV (MGAT4A and MGAT4B) has been reported in pancreatic cancer (27).

*O*-GlcNAcylation is a dynamic and reversible PTM in which a single GlcNAc molecule attaches to the serine (Ser) or threonine (Thr) residue of cytoplasmic and nuclear proteins (28). This modification is catalyzed by *O*-GlcNAc transferase (OGT) and removed by *O*-GlcNAcase (OGA). *O*-GlcNAcylation has been detected on numerous regulatory proteins, including p53 (29), HIF-1α (30), β-catenin (31), and glucose-6-phosphate dehydrogenase (G6PD) (32), all of which play key roles in cellular metabolism, proliferation, and oncogenic signaling. Increased *O*-GlcNAcylation has been observed in pancreatic tumors and is linked to activation of the NF-κB signaling pathway (33). Additionally, knockdown of OGT in breast and liver cancer cell lines has been shown to reduce cell motility (31, 34), further supporting the role of *O*-GlcNAcylation in regulating tumorigenicity, including cancer cell migration and invasiveness (35). Because these pathways draw on the same UDP-GlcNAc pool, alterations in HA metabolism could rewire *N*-glycan branching and *O*-GlcNAcylation through metabolic competition and signaling crosstalk.

In this study, we generated HAS2 KO and CD44 KO cell lines to examine how HA regulates intracellular UDP-GlcNAc levels and its effects on cellular functions. Our results showed that HA deficiency significantly increased intracellular UDP-GlcNAc, which enhanced cellular *N*-glycosylation and *O*-GlcNAcylation. Adding exogenous HA reversed these effects in a CD44-dependent manner. These findings suggest that HA serves as a structural component of the ECM and as a metabolic regulator, influencing cellular biology and function. Consequently, this study offers new insights into the metabolic role of HA in cancer biology and emphasizes its potential as a therapeutic target in tumor progression.

## Results

### Deficiency of HAS2 suppressed HA levels

**Figure 1:**
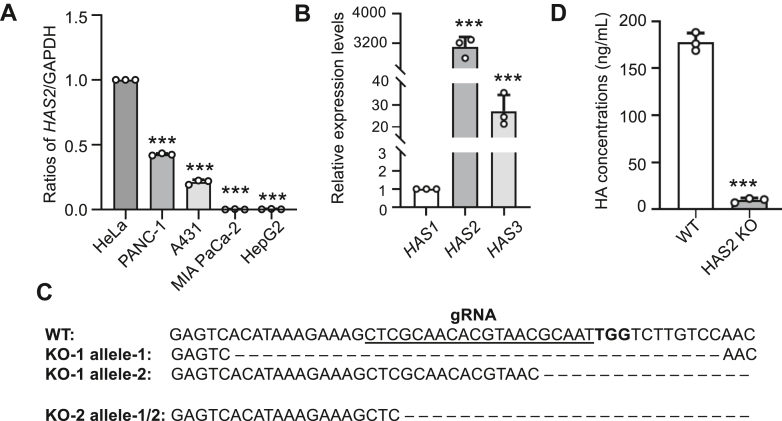
**Establishment of HAS2 KO cells and confirmation of HA synthesis.***A*, comparison of HAS2 expression levels among different cell lines: HeLa (cervical adenocarcinoma cell line), A431 (epidermoid carcinoma cell line), PANC-1 (pancreatic ductal adenocarcinoma cell line), MIA PaCa-2 (pancreatic carcinoma cell line), and HepG2 (hepatocellular carcinoma cell line). The *HAS2* mRNA level in HeLa was normalized to 1.0. All values are presented as mean ± SD from three independent experiments, based on one-way ANOVA with Tukey’s *post hoc* analysis. ∗∗∗*p* < 0.001. *B*, comparison of *HAS1*, *HAS2*, and *HAS3* expression levels in HeLa cells. The *HAS1* mRNA level in HeLa was normalized to 1.0. All values are presented as mean ± SD from three independent experiments, based on one-way ANOVA with Tukey’s *post hoc* analysis. ∗∗∗*p* < 0.001. *C*, Sanger sequencing was used to analyze exon 2 of the target sequence in HeLa WT and HAS2 KO cells with the canonical SpCas9 PAM sequence (TGG) highlighted in bold. *D*, WT and HAS2 KO cells were cultured in serum-free medium, and the conditioned medium was collected after 24 h to measure HA concentration. Statistical significance was determined from three independent experiments using an unpaired Student’s *t* test, with *p* values indicated as ∗∗∗*p* < 0.001.

HA is synthesized by HAS, which exists in three distinct types, from HAS1 to HAS3. Mice lacking HAS2 are embryonically lethal, highlighting the essential role of HAS2. Therefore, we performed qPCR analysis to examine HAS2 expression across five cell lines and found it was most highly expressed in HeLa cells (Fig. 1*A*). As a result, we selected HeLa cells with high *HAS2* gene expression as the model cells for this study. Additionally, qPCR analysis of *HAS1*, *HAS2*, and *HAS3* in HeLa cells showed that *HAS2* expression was approximately three orders of magnitude higher than *HAS1* and *HAS3*, further confirming that *HAS2* is the main HAS isoform in this cell line (Fig. 1*B*).

To investigate the impact of HAS2 deficiency on cellular and biochemical processes, we employed the CRISPR/Cas9 system to generate a HAS2 knockout (KO) cell line. Sanger sequencing confirmed the genetic alterations, revealing significant deletions in both alleles that caused frameshift mutations with premature stop codons (Fig. 1*C*). Additionally, the culture supernatant of WT cells contained 178.1 ng/ml of HA, while HA production in HAS2 KO cells was significantly reduced to 9.8 ng/ml (Fig. 1*D*). Therefore, HAS2 is a crucial enzyme for HA synthesis in HeLa cells.

### HAS2 KO reduced cellular proliferative and migratory capacity

**Figure 2:**
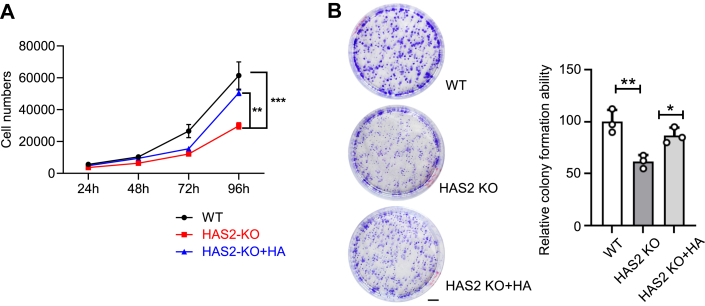
**Effects of HAS2 KO and exogenous HA on cell proliferation and colony formation.***A*, cell proliferation was measured by directly counting cells. HAS2 KO cells were cultured in medium with or without HA at a concentration of 200 ng/ml. Cell numbers were counted at specific time points using a hemocytometer. Data are shown as mean ± SD from three independent experiments and analyzed with two-way ANOVA. ∗∗*p* < 0.01; ∗∗∗*p* < 0.001. *B*, colony formation was assessed with WT set at 100%. Scale bar: 5 mm. All values are presented as mean ± SD from three independent experiments, analyzed with one-way ANOVA and Tukey’s *post hoc* test. ∗*p* < 0.05; ∗∗*p* < 0.01.

Although HA biosynthesis is often elevated in cancer, its specific contribution to cancer cell function remains unclear. Therefore, cell counting and colony assays were performed to investigate the role of HA in proliferation. As shown in Figure 2*A*, cell proliferation was significantly reduced in HAS2 KO cells, which was restored by adding HA. Furthermore, compared to WT cells, KO cells exhibited significantly smaller colonies and fewer numbers (Fig. 2*B*), which was partially rescued by HA. These results suggest that HA synthesized by HAS2 has a positive regulatory effect on cancer cell proliferation and survival.

**Figure 3:**
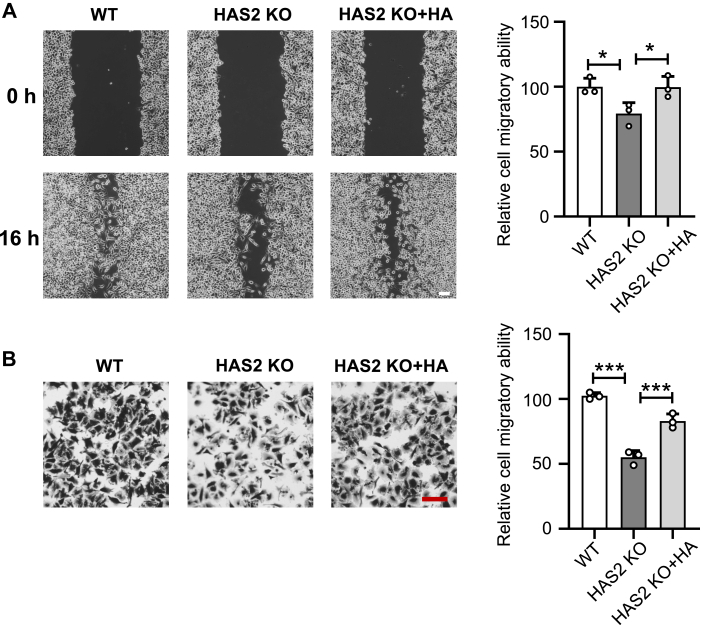
**Effects of HAS2 KO and exogenous HA on cell migration ability.** The effect of HA on cell migration was assessed using a wound-healing assay and a Transwell assay, as described in “Experimental procedures.” Representative images for each cell type are shown after migration in both assays. *A*, WT or HAS2 KO cells treated with or without HA were cultured for 16 h following scratching in the wound-healing assay. The migration ability of WT cells was set at 100%. All values are expressed as mean ± SD from three independent experiments, analyzed with one-way ANOVA and Tukey’s *post hoc* test. ∗*p* < 0.05. *B*, cell migration was measured using the Transwell assay. In the HA-treated group, cells were pre-cultured in medium containing 200 ng/ml HA for 48 h before migration assessment over a 4-h period. The migration of WT cells was set at 100%. All data are presented as mean ± SD from three independent experiments, analyzed with one-way ANOVA and Tukey’s *post hoc* test. ∗∗∗*p* < 0.001. Scale bar: 50 μm.

The reduced migratory ability of HAS2 KO cells is a key factor in cancer aggressiveness, mainly due to changes in cell proliferation and movement. Therefore, we investigated how HA biosynthesis influences cell migration using the wound-healing assay (Fig. 3*A*) and the Transwell assay (Fig. 3*B*). Both assays consistently demonstrated a significant decrease in the migratory capacity of HAS2 KO cells, which aligns with the reduced cell proliferation seen in Figure 2. Additionally, adding HA to the HAS2 KO cells restored their migration to the level of WT cells. Furthermore, the decreased migratory ability of HAS2 KO cells was significantly restored by reintroducing *HAS2* into the HAS2 KO cells, known as the rescue cells (RES), using wound-healing assays (Supplementary data, Fig. S1). These results indicate a strong link between HA produced by HAS2 and cell migration.

### The levels of UDP-GlcNAc were increased in HAS2 KO cells

Sugar nucleotides are essential donor substrates for glycosyltransferases involved in glycan chain synthesis. For example, UDP-GlcNAc serves as the donor for HA, *N*-linked glycans, and *O*-GlcNAcylation. UDP-GlcA serves as another donor for HA. UDP-GlcNAc is produced *via* the hexosamine biosynthetic pathway (HBP), which is part of glucose metabolism (36). It functions as a substrate for protein glycosylation and as a precursor for other nucleotide sugars. UDP-GlcA is generated from UDP-glucose by UDP-glucose dehydrogenase (37). Notably, the accumulation of UDP-Glc within cells has been shown to inhibit epithelial–mesenchymal transition (EMT) (38). These findings emphasize that changes in the levels of one nucleotide sugar can affect the biosynthesis and function of others, highlighting a coordinated regulatory network among sugar nucleotide pathways.

**Figure 4:**
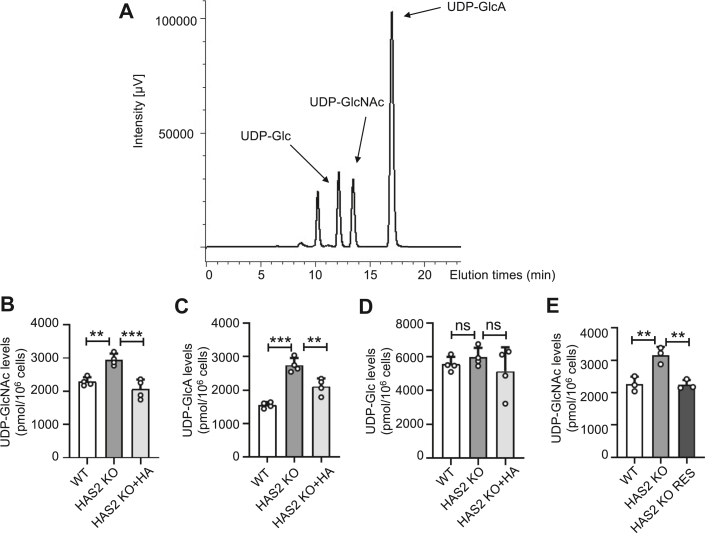
**Effects of HAS2 KO and exogenous HA on nucleotide sugar levels.** Cells were collected with ice-cold 70% ethanol, and the supernatant was purified *via* ion-pair solid-phase extraction. Frozen samples were dissolved in 100 μl of Milli-Q water, and 10 μl of each sample was injected into the column. Each peak was identified by comparing its retention time with that of the standards (*A*). Using calibration curves and normalization to cell number, nucleotide sugar concentrations were determined for UDP-GlcNAc (*B*), UDP-GlcA (*C*) and UDP-Glc (*D*). HAS2 KO cells were cultured for 48 h in medium supplemented with or without 200 ng/ml of HA. All values are from four independent experiments, using one-way ANOVA with Tukey’s *post hoc* test, expressed as mean ± SD. ∗∗*p* < 0.01; ∗∗∗*p* < 0.001 or indicated as no significance (ns). *E*, comparison of intracellular UDP-GlcNAc levels among WT, HAS2 KO, and HAS2 KO RES cells, which were rescued with *HAS2* into the HAS2 KO cells. Data are presented as mean ± SD from three independent experiments. Statistical significance was determined by one-way ANOVA followed by Tukey’s *post hoc* test. ∗∗*p* < 0.01.

Therefore, we analyzed changes in HAS2 KO cells. Figure 4*A* displays the HPLC elution profile of various nucleotide sugars. The levels of nucleotide sugars were determined from peak areas and normalized to the total cell count (Fig. 4, *B*–*D*). UDP-GlcNAc levels were notably higher in HAS2 KO cells compared to WT cells. Interestingly, when external HA was added to HAS2 KO cells, UDP-GlcNAc levels returned to normal, similar to WT cells (Fig. 4*B*). UDP-GlcA levels also increased significantly in HAS2 KO cells. Interestingly, adding exogenous HA slightly reduced these levels. Meanwhile, there were no significant changes in other sugars like UDP-Glc (Fig. 4*D*). Furthermore, the increased UDP-GlcNAc levels in HAS2 KO cells were effectively restored to levels similar to those of WT cells in the RES (Fig. 4*E*). These findings suggest that extracellular HA influences intracellular metabolic regulation.

### HAS2 KO increased levels of GlcNAc-branched *N*-glycans and *O*-GlcNAcylation

**Figure 5:**
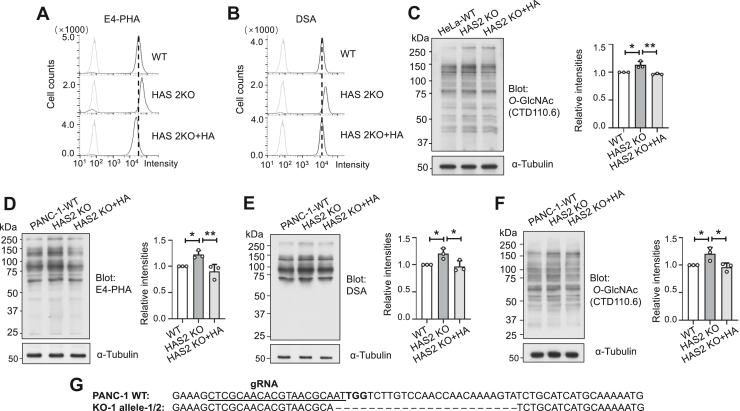
**Alterations in GlcNAc-branched N-glycans and O-GlcNAcylation in HAS2 KO in HeLa and PANC-1 cells.** The WT HeLa cells and HAS2 KO cells were cultured for 48 h in medium supplemented with or without 200 ng/ml of HA, then analyzed by flow cytometry using E4-PHA (*A*) and DSA (*B*), which preferentially recognize the bisecting GlcNAc and GlcNAcβ1,4-mannosylated *N*-glycans, respectively. *C*, the same amounts of cell lysates were subjected to a 7.5% SDS-PAGE gel and probed with anti-*O*-GlcNAc antibody. α-Tubulin was used as a loading control. The ratio of *O*-GlcNAc *versus* α-Tubulin with the WT was set as 1.0. All data were obtained from three independent experiments and analyzed by one-way ANOVA with Tukey’s multiple comparison test and displayed as the mean ± SD. ∗*p* < 0.05, ∗∗*p* < 0.01. The same amounts of cell lysates from WT PANC-1 cells and HAS2 KO cells treated with or without 200 ng/ml of HA were subjected to a 7.5% SDS-PAGE gel and stained with E4-PHA lectin (*D*), DSA lectin (*E*), and anti-*O*-GlcNAc antibody (*F*). α-Tubulin was used as a loading control. The ratio of E4-PHA to α-Tubulin, DSA to α-Tubulin and *O*-GlcNAc to α-Tubulin with the WT was set as 1.0. All data were obtained from three independent experiments, analyzed using one-way ANOVA with Tukey’s multiple comparison test and shown as the mean ± SD. ∗*p* < 0.05, ∗∗*p* < 0.01. *G*, Sanger sequencing was used to analyze exon 2 of the target sequence in PANC-1 WT and HAS2 KO cells.

The changes in branched *N*-glycans in HAS2 KO cells were examined using flow cytometry analysis. E4-PHA, which preferentially detects bisected *N*-glycans produced by GnT-III (39), and DSA, which binds to β1,4-GlcNAc-branched *N*-glycans created by GnT-IV (40), were used to assess the structural modifications of *N*-glycans. Flow cytometry results showed a significant increase in reactivity with E4-PHA and DSA in HAS2 KO cells compared to WT cells (Fig. 5, *A* and *B*). Additionally, changes in *O*-GlcNAcylation in HAS2 KO cells were analyzed using Western blot. As shown in Figure 5*C*, *O*-GlcNAcylation levels of proteins around the 70 to 150 kDa range on SDS-PAGE were elevated. Consistent with the data in Figure 4, these increases were normalized when HA was added to the HAS2 KO cells. Notably, similar findings were observed in PANC-1 pancreatic cancer cells (Fig. 5, *D*–*F*), and these results were further supported by the quantitative analyses shown in the figure panels.

To further investigate whether these changes were solely due to elevated UDP-GlcNAc levels or also involved transcriptional regulation of related glycogenes, we analyzed the expression of key enzymes by qPCR (Fig. S2). The expression levels of *MGAT4A* decreased, while *MGAT4B* expression increased in HAS2 KO cells compared to WT cells. Additionally, *OGT* levels were elevated, whereas *OGA* levels decreased. Furthermore, we conducted Western blot analysis of OGT and OGA in HAS2 KO cells and in HAS2 KO cells rescued with FLAG-HAS2 (HAS2 KO RES cells) to confirm the *O*-GlcNAcylation changes observed in Figure 5. Consistently, OGT protein levels increased in HAS2 KO cells and decreased in HAS2 KO RES cells (Fig. S3). Meanwhile, OGA protein expression showed no significant differences among these cells, which is somewhat contradictory to the mRNA data (Fig. S2*B*). Although the underlying mechanisms require further study, these results indicate that the increased *N*-glycan branching and *O*-GlcNAcylation in HAS2 KO cells are not only due to elevated UDP-GlcNAc levels but also involve transcriptional regulation of the enzymes responsible for these modifications.

From these observations, we can infer that cells may sense extracellular HA levels to regulate intracellular UDP-GlcNAc levels, which in turn affect the biosynthesis of branched *N*-glycans and *O*-GlcNAcylation.

### CD44 KO reduced cellular proliferative and migratory abilities

**Figure 6:**
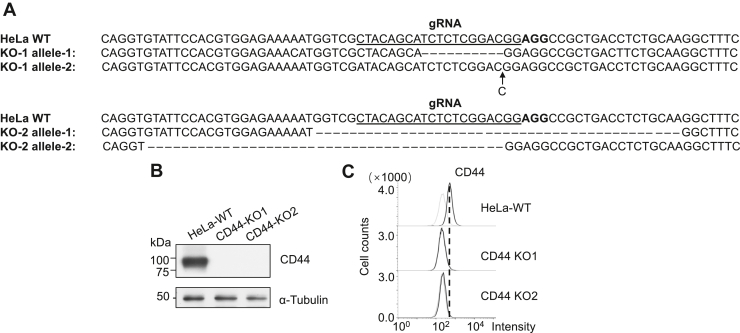
**Establishment of CD44 KO cells.***A*, Sanger sequencing was conducted to verify the genomic alterations in the CD44 KO cells with the canonical SpCas9 PAM sequence (AGG) highlighted in bold. *B*, equal amounts of cell lysates were subjected to Western blotting using an anti-CD44 antibody to assess CD44 expression in WT and CD44 KO cells, with α-Tubulin serving as a loading control. *C*, the expression levels of CD44 protein on the cell surface were analyzed by flow cytometry.

**Figure 7:**
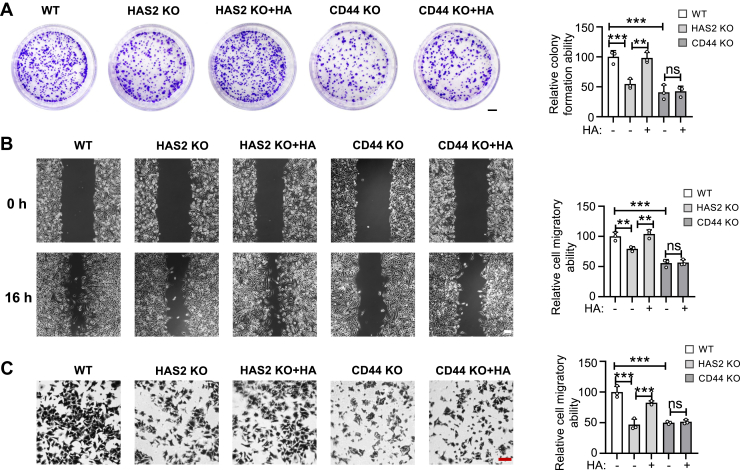
**Comparison of cell proliferation and migration capabilities in HAS2 KO and CD44 KO cells, with or without exogenous HA.** The Transwell assay, as described in the “Experimental procedures,” was used to examine cell migration. *A*, representative images for each indicated cell type are shown after the colony formation assay. HAS2 KO or CD44 KO cells were cultured in medium with or without HA at a concentration of 200 ng/ml. Quantitative analysis of colony formation involved counting colonies from three random fields. All values represent the mean ± SD. ∗∗*p* < 0.01; ∗∗∗*p* < 0.001 or no significance (ns). The proliferation ability of WT cells was set at 100%. *B*, representative images for each indicated cell are displayed after the wound-healing assay. Cells were cultured for 16 h following scratching in the wound-healing assay. In the HA-treated group, medium containing 200 ng/ml HA was added immediately after scratching, and cells were cultured for an additional 16 h. The *right panel* provides a quantitative analysis of cell migration. Data were obtained by counting the migrated cells from three randomly selected fields. The migration of WT cells was set at 100%. All values represent the mean ± SD. ∗∗*p* < 0.01; ∗∗∗*p* < 0.001 or no significance (ns). *C*, representative images for each indicated cell are shown after the Transwell assay. In this Transwell assay, migration was measured after 4 h. In the HA-treated group, cells were pre-cultured in medium containing 200 ng/ml HA for 48 h before migration assessment over a 4-h period. The right panel presents a quantitative analysis of cell migration. The migration of WT cells was set at 100%. Data were obtained by counting migrated cells from three random fields. All values represent the mean ± SD. ∗∗∗*p* < 0.001 or no significance (ns).

CD44 is a primary cell surface receptor for HA and a well-established cancer biomarker. To determine whether HA's biological effects observed above are mediated through CD44, we generated a CD44 KO cell line using the CRISPR/Cas9 system. The KO efficiency was confirmed by genomic sequence analysis (Fig. 6*A*), Western blot (Fig. 6*B*), and flow cytometry analysis (Fig. 6*C*). Through colony formation (Fig. 7*A*), wound-healing (Fig. 7*B*), and Transwell assays (Fig. 7*C*), we found that CD44 KO cells exhibited significantly reduced migratory and clonogenic capabilities compared to WT cells. Importantly, adding exogenous HA did not restore these phenotypes, indicating that HA’s effects on cell migration and proliferation depend heavily on CD44-mediated signaling.

### CD44 KO-reduced levels of UDP-GlcNAc, GlcNAc-branched *N*-glycans, and *O*-GlcNAcylation, and these were not restored by exogenous HA

**Figure 8:**
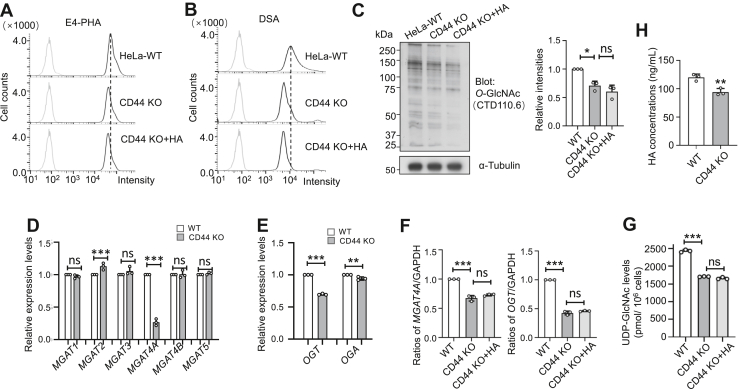
**Effects of CD44 on the expression of GlcNAc-branched *N*-glycans and *O*-GlcNAcylation, and related glycosyltransferase genes.** The CD44 KO HeLa cells were cultured for 48 h in medium supplemented with or without 200 ng/ml of HA, then incubated with lectins to assess glycosylation changes on the cell surface using flow cytometric analysis, such as E4-PHA (*A*) and DSA (*B*) lectin. *C*, the same amounts of cell lysates were subjected to a 7.5% SDS-PAGE gel and stained with anti-*O*-GlcNAc antibody. α-Tubulin was used as a loading control. The ratio of *O*-GlcNAc *versus* α-Tubulin with the WT was set as 1.0. All data were analyzed using a one-way ANOVA test with Tukey’s multiple comparison test and are displayed as the mean ± SD. ∗*p* < 0.05 or no significance (ns). All data are representative of three independent experiments. *D*, mRNA levels of several *N*-acetylglucosaminyltransferases involved in synthesizing GlcNAc-branched *N*-glycans were determined using qPCR. GAPDH was used as an internal control, and values were normalized to WT cells (set as 1.0). Statistical significance was assessed from three independent experiments using the unpaired Student’s *t* test, with *p*-values indicated as ∗∗∗*p* < 0.001 or no significance (ns). *E*, mRNA levels of *OGT* and *OGA* were determined using qPCR. GAPDH was used as an internal control, and values were normalized to WT cells (set as 1.0). Statistical significance was assessed from three independent experiments using the unpaired Student’s *t* test, with *p* values indicated as ∗∗∗*p* < 0.001, ∗∗*p* < 0.01. *F*, mRNA levels of *MGAT4A* and *OGT* expression in CD44 KO cells with or without HA supplement were determined using qPCR. *GAPDH* was used as an internal control, and values were normalized to WT cells (set as 1.0). Statistical significance was assessed from three independent experiments using the unpaired Student’s *t* test, with *p*-values indicated as ∗∗∗*p* < 0.001 or no significance (ns). *G*, comparison of intracellular UDP-GlcNAc levels among WT, CD44 KO and CD44 KO cells treated with HA. Quantification was performed as described in Figure 4. Data are presented as mean ± SD from three independent experiments. All values reflect one-way ANOVA with Tukey’s *post hoc* analysis, expressed as the mean ± SD. ∗∗∗*p* < 0.001 or no significance (ns). *H*, WT and CD44 KO cells were cultured in serum-free medium, and the conditioned medium was collected after 24 h to measure HA concentration. Statistical significance was determined from three independent experiments using an unpaired Student’s *t* test, with *p*-values indicated as ∗∗*p* < 0.01.

To further investigate the effect of CD44 KO on protein glycosylation, we measured levels of GlcNAc-branched *N*-glycans and *O*-GlcNAcylation. Flow cytometry analysis using E4-PHA and DSA showed reductions in GlcNAc-branched *N*-glycans on the cell surface in CD44 KO cells compared to WT cells (Fig. 8, *A* and *B*). Western blot analysis also indicated significant decreases in *O*-GlcNAcylation levels (Fig. 8*C*). Notably, adding exogenous HA did not reverse these changes, suggesting that the altered glycosylation profile likely depends on CD44 expression and cannot be restored by HA alone without its primary receptor. Additionally, qPCR analysis of the GnT family revealed a significant decrease in *MGAT4A* mRNA levels, while *MGAT2* showed a slight increase in CD44 KO cells compared to WT cells (Fig. 8*D*). CD44 KO cells also showed a notable reduction in *OGT* and *OGA* expression levels, as determined by qPCR (Fig. 8*E*). Importantly, exogenous HA did not restore the decreases in *MGAT4A* and *OGT* expression in CD44 KO cells, further supporting the necessity of CD44 for HA-mediated regulation (Fig. 8*F*). The specific mechanisms behind these transcriptional changes warrant further investigation.

Additionally, intracellular UDP-GlcNAc levels were measured using HPLC. These levels were significantly lower in CD44 KO cells compared to WT, and this decrease could not be reversed by exogenous HA (Fig. 8*G*). Furthermore, HA content was lower in CD44 KO cells than in WT cells (Fig. 8*H*), consistent with the decreased intracellular UDP-GlcNAc levels observed in these cells. This may partly explain the reduced expression of GlcNAc-branched N-glycans and O-GlcNAcylation in CD44 KO cells. Along with the results shown in Figure 7, these findings further support the important role of HA in controlling cellular behaviors, functioning in a CD44-dependent manner.

### CD44 KO reduced GFAT expression and UDP-GlcNAc, accompanied by decreased cell migration

**Figure 9:**
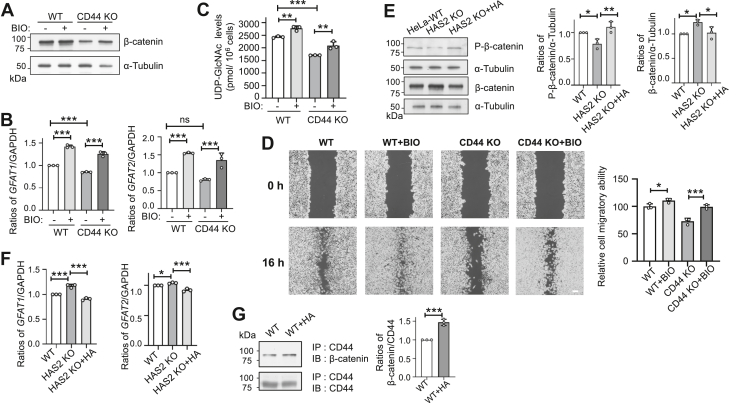
**Effects of CD44 KO on the Wnt signaling, which regulates the expression of UDP-GlcNAc and *GFAT,* as well as migratory capacity.***A*, WT or CD44 KO HeLa cells were cultured with or without 2 μM BIO. Equal amounts of protein from these cells were analyzed by Western blot using the indicated antibodies. α-Tubulin served as a loading control. *B*, mRNA levels of GFAT1 and GFAT2 were measured *via* qPCR in WT and CD44 KO HeLa cells treated with or without BIO. GAPDH was used as an internal control. All values were normalized to GAPDH levels, with the ratio of WT without BIO treatment set as 1.0. Data are shown as mean ± SD from three independent experiments. *p* values were determined by one-way ANOVA with Tukey’s *post hoc* test. ∗∗∗*p* < 0.001 or noted as no significance (ns). *C*, effects of BIO on intracellular UDP-GlcNAc levels in WT and CD44 KO cells. Quantification followed the method in Figure 4. Data are expressed as mean ± SD from three experiments. All values are from one-way ANOVA with Tukey’s *post hoc* analysis, shown as mean ± SD. ∗∗∗*p* < 0.001, ∗∗*p* < 0.01. *D*, effects of BIO on cell migration in WT and CD44 KO cells, with representative images after the wound-healing assay. Quantitative analysis was based on counting migrated cells in three randomly selected fields. All data are mean ± SD, with WT without BIO set as 100. ∗*p* < 0.05; ∗∗∗*p* < 0.001. *E*, equal protein amounts from WT or HAS2 KO cells treated with or without HA were analyzed by Western blot with β-catenin and P-β-catenin antibody. α-Tubulin served as a loading control. The ratios of P-β-catenin to α-Tubulin and β-catenin to α-Tubulin in WT cells were set as 1.0. Data are shown as mean ± SD from three independent experiments. *p*-values were calculated by one-way ANOVA with Tukey’s *post hoc* test. ∗*p* < 0.05, ∗∗*p* < 0.001. *F*, mRNA levels of GFAT1 and GFAT2 measured by qPCR in WT and HAS2 KO cells treated with or without HA. GAPDH was the internal control. Values were normalized to GAPDH, with the WT without HA ratio set at 1.0. Data are shown as mean ± SD from three independent experiments. *p*-values were calculated by one-way ANOVA with Tukey’s *post hoc* test. ∗*p* < 0.05; ∗∗∗*p* < 0.001. *G*, equal amounts of proteins obtained from WT HeLa cells treated with or without HA were immunoprecipitated using Ab-Capcher with an anti-CD44 antibody. The immunoprecipitates were then Western blotted with an anti-β-catenin antibody. The ratio of β-catenin to CD44 with the WT was set as 1.0. Statistical significance was determined from three independent experiments using an unpaired Student’s *t* test, with *p*-values indicated as ∗∗∗*p* < 0.001.

Previous studies have demonstrated that CD44 functions as a positive regulator of the Wnt receptor complex, and β-catenin is closely associated with cell proliferation, migration, and invasion (41). Therefore, we first measured the expression levels of β-catenin using Western blot analysis. The results indicated that intracellular β-catenin levels were significantly decreased after CD44 KO (Fig. 9*A*). To determine whether Wnt signaling influences UDP-GlcNAc levels, we first examined the expression levels of *GFAT1* and *GFAT2*, the rate-limiting enzymes in the HBP (42). We found that both were significantly downregulated in CD44 KO cells compared to WT (Fig. 9*B*). To further confirm whether Wnt signaling affects the expression of *GFAT1* and *GFAT2*, we treated cells with BIO, a well-known activator of the Wnt pathway (43, 44). BIO works by inhibiting GSK3β, which disrupts the β-catenin destruction complex and prevents β-catenin phosphorylation. As a result, β-catenin avoids proteasomal degradation, accumulates in the cytoplasm, and then translocates into the nucleus to activate target gene transcription (45). qPCR analysis showed that BIO treatment significantly increased *GFAT1* and *GFAT2* expression levels in CD44 KO cells (Fig. 9*B*). Consistent with these transcriptional changes, intracellular UDP-GlcNAc levels were notably lower in CD44 KO cells, but this reduction was rescued by BIO treatment (Fig. 9*C*). These results suggest that CD44 may influence glycosylation patterns through Wnt signaling, with *GFAT1/2* serving as key downstream effectors in this regulatory pathway.

To investigate the role of Wnt signaling in cell behavior, we performed a wound-healing assay in WT and CD44 KO cells (Fig. 9*D*). CD44 KO significantly reduced cell migration. Treatment with BIO slightly enhanced migratory ability in WT cells, suggesting that β-catenin promotes cell migration. Similarly, BIO treatment significantly increased cell migration in CD44 KO cells.

To further clarify how HA interacts with β-catenin signaling, we conducted analyses in HAS2 KO cells. Western blot results showed a significant decrease in phosphorylated β-catenin levels in HAS2 KO cells, along with an increase in total β-catenin. Adding exogenous HA restored these changes to WT levels (Fig. 9*E*). Similarly, the expression of GFAT1 and GFAT2 increased in HAS2 KO cells and decreased after HA treatment (Fig. 9*F*). To determine whether CD44 interacts with β-catenin, we conducted co-immunoprecipitation experiments with or without exogenous HA. Western blot analysis showed that CD44 indeed co-precipitated with β-catenin, and this interaction was enhanced by HA supplementation (Fig. 9*G*), suggesting that HA promotes the association between CD44 and β-catenin. Although the mechanism behind the upregulation of β-catenin in HAS2 KO cells remains unclear, these results support the idea that HA regulates UDP-GlcNAc synthesis and cellular functions *via* the CD44-dependent β-catenin pathway. Additionally, these findings indicate that β-catenin signaling is essential for CD44-mediated cell migration, and activating this pathway can partially compensate for the migration defects caused by CD44 deficiency. Overall, this study demonstrates that extracellular HA influences intracellular UDP-GlcNAc levels by regulating GFAT expression, which in turn affects cellular glycosylation through CD44.

### CD44 overexpression did not reduce elevated UDP-GlcNAc levels or impaired cell migration in HAS2 KO cells

**Figure 10:**
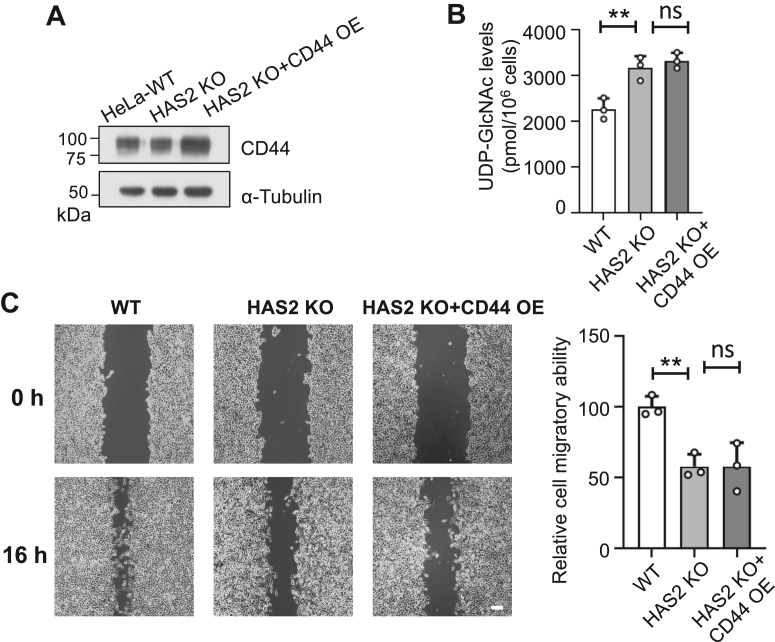
**Effects of CD44 overexpression on UDP-GlcNAc levels and migratory capacity in HAS2 KO cells.***A*, the same amounts of cell lysates were subjected to Western blotting using an anti-CD44 antibody to assess CD44 expression levels in WT, HAS2 KO, and HAS2 KO cells overexpressing CD44 (HAS2 KO + CD44 OE). α-Tubulin served as a loading control. *B*, comparison of intracellular UDP-GlcNAc levels among WT, HAS2 KO, and HAS2 KO + CD44 OE cells. Quantification was performed as described in Figure 4. Data are presented as mean ± SD from three independent experiments. All values reflect one-way ANOVA with Tukey’s *post hoc* analysis, expressed as mean ± SD. ∗∗*p* < 0.01 or no significance (ns). *C*, WT, HAS2 KO, and HAS2 KO + CD44 OE cells were cultured for 16 h following scratching in the wound-healing assay. The migration ability of WT cells was set at 100%. All values are expressed as mean ± SD from three independent experiments and analyzed with one-way ANOVA and Tukey’s *post hoc* test. ∗∗*p* < 0.01 or no significance (ns).

To determine whether increasing CD44 levels could rescue the phenotypes observed in HAS2 KO cells, we overexpressed *CD44* in these cells and analyzed cellular UDP-GlcNAc levels and cell migration. Western blot confirmed a significant increase in CD44 expression after overexpression (Fig. 10*A*). However, the overexpression did not decrease the elevated UDP-GlcNAc levels in HAS2 KO cells (Fig. 10*B*). It also failed to restore the impaired migration phenotype in these cells (Fig. 10*C*). These results suggest that simply increasing CD44 is insufficient to compensate for the loss of HAS2, and that the interaction between HA and CD44—rather than CD44 levels alone—is critical for regulating UDP-GlcNAc metabolism and cell behavior.

## Discussion

**Figure 11:**
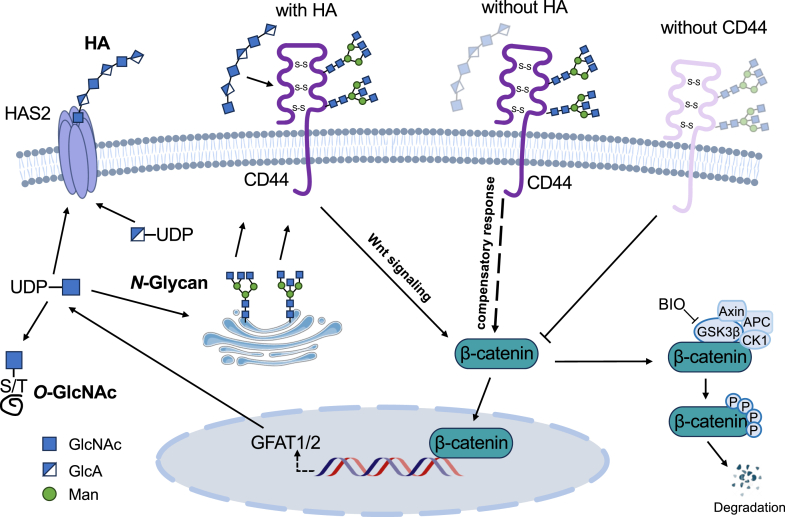
**Proposed molecular mechanism of how HA affects changes in glycosylation and cellular functions through Wnt signaling.** This study demonstrates that HA regulates cellular glycosylation and related functions through CD44 *via* the β-catenin signaling pathway. HA is mainly produced by HAS2 using UDP-GlcNAc and UDP-GlcA as substrates. When HA binds to CD44, it may stabilize β-catenin by preventing its phosphorylation and subsequent degradation. Stabilized β-catenin then accumulates in the cytoplasm and moves to the nucleus, where it influences the transcription of GFAT1/2. As a result, intracellular UDP-GlcNAc levels increase, leading to changes in *N*- and *O*-linked glycosylation patterns. These glycosylation changes can affect various cellular behaviors, including proliferation and migration. Interestingly, cells seem to detect extracellular HA levels. In the absence of HA, meaning no HA association with CD44, a compensatory response is triggered, resulting in increased β-catenin accumulation and upregulation of GFAT1/2, which enhances UDP-GlcNAc production. However, in the absence of CD44, cells cannot sense HA, thereby disrupting this signaling pathway. As a result, β-catenin is no longer stabilized, its levels decrease, and the compensatory upregulation of GFAT1/2 cannot occur. Because of the central role of the CD44-β-catenin axis in coordinating glycosylation and cell behavior, targeting this pathway could provide new insights into cancer biology. The dotted line indicates that the exact molecular mechanism remains unclear.

Increasing evidence suggests that HA is often associated with enhanced migratory and invasive capacities in cancer cells (4, 46, 47). To clarify the underlying molecular mechanisms, we established HAS2 KO and CD44 KO cell lines in HeLa cells. The HAS2 KO disrupts HA synthesis and significantly reduces cell proliferation and migration, accompanied by elevated intracellular levels of UDP-GlcNAc, increased *N*-glycan branching, and enhanced *O*-GlcNAcylation. These effects were also observed in another cell line, PANC-1, a human pancreatic ductal adenocarcinoma cell line that expresses relatively higher levels of HAS2. These alterations were reversed by adding HA at physiological concentrations, highlighting the importance of HA availability in maintaining cell behavior and glycosylation (48, 49, 50). Notably, these HA-dependent effects were not observed in CD44 KO cells, suggesting that the regulatory role of HA depends on CD44-mediated signaling (51, 52). Further analysis revealed that CD44 KO suppressed Wnt signaling (53, 54, 55, 56), specifically decreasing β-catenin expression levels, which resulted in the downregulation of GFAT1 and GFAT2, two enzymes involved in UDP-GlcNAc biosynthesis (57). Together, these findings support a mechanistic model in which extracellular HA regulates glycosylation metabolism and cancer cell migration through CD44, mediated by the Wnt signaling pathway (Fig. 11). Moreover, β-catenin is directly *O*-GlcNAcylated, and this modification is thought to stabilize β-catenin and enhance its nuclear transcriptional activity (58, 59). Therefore, an increase in UDP-GlcNAc availability downstream of the CD44-GFAT axis may enhance Wnt/β-catenin signaling by promoting *O*-GlcNAcylation-mediated stabilization of β-catenin.

It is known that the cellular steady-state level of UDP-GlcNAc is influenced not only by the transcriptional regulation of HBP enzymes but also by changes in its utilization in glycan synthesis. The main consumers of UDP-GlcNAc are likely *N*-glycans, *O*-GlcNAcylation, and HA synthesis. Therefore, a reduction in HA synthesis alone probably cannot explain the significant increase in UDP-GlcNAc levels observed in HAS2 KO cells. Additionally, the expression of GFAT1/2 was considerably increased in HAS2 KO cells, and this increase was completely reversed by exogenous HA (Fig. 9*F*). This transcriptional response supports the idea that the restoration of UDP-GlcNAc levels is linked to HA depletion-induced regulation of HBP gene expression, rather than a decrease in substrate consumption for HA synthesis. Although the exact role of HA consumption in cellular UDP-GlcNAc levels remains uncertain, the current findings suggest that the CD44-dependent signaling mainly regulates UDP-GlcNAc levels through transcriptional control of HBP enzymes, rather than through consumption during HA synthesis.

The reduction of cell proliferation and migration in HAS2 KO cells can be reversed by adding HA, emphasizing the critical role of HA in cellular functions. This finding aligns with previous research showing that adding 4-Methylumbelliferone (4-MU), a HA inhibitor, decreases cell proliferation and migration in cancer cells (60, 61, 62). The inhibition caused by 4-MU involves two main mechanisms: first, it competes with UDP-glucuronosyltransferase (UGT) to lower UDP-GlcA levels (63); second, it reduces mRNA expression of *HAS2* and *HAS3* (64) as well as the expression of UDP-glucose pyrophosphorylase and UDP-glucose 6-dehydrogenase (UGDH) (65). As a result, in pancreatic cancer, adding 4-MU can decrease the migratory ability and slow disease progression (66), while also decreasing cell proliferation in melanoma (61). These previous reports support our findings regarding the impact of HA synthase deficiency on cancer cell proliferation and migration, which are associated with malignancy. Targeting HA biosynthesis not only highlights the crucial role of HA in tumor progression but also presents significant potential as a precise therapeutic target for cancer intervention.

Significantly, these regulatory effects depend not only on the structural presence of HA in the ECM but also on its ability to activate intracellular signaling pathways. As a candidate for sensing HA, CD44, a well-known receptor for HA and a recognized biomarker for cancer stem cells (14, 67, 68, 69), can be considered. Although the detailed mechanisms of CD44 sensing for HA remain to be further investigated, we can hypothesize a conceptual model: under normal conditions with an appropriate HA concentration, CD44 molecules may be sufficiently occupied by HA, helping to maintain cellular homeostasis; when HA levels decrease, some of CD44 molecules become free of HA, which may lead to altered downstream signaling for compensatory responses (Fig. 11). The interaction between HA and CD44 induces EMT (70), a process in which epithelial cells acquire mesenchymal characteristics. EMT is associated with the loss of cell adhesion and the gain of motility related to cancer malignancy (71, 72). In addition to its well-established role in promoting EMT *via* TGF-β and EMT-associated transcription factors such as Snail and Twist (72, 73), CD44 also modulates signaling pathways directly linked to malignant phenotypes (74, 75). Furthermore, previous studies have shown a link between EMT and intracellular UDP-Glc levels. UGDH, which converts UDP-Glc to UDP-GlcA, promotes EMT by stabilizing *SNAI1* mRNA, while accumulation of UDP-Glc inhibits this process (38). In this study, higher levels of UDP-GlcNAc and UDP-GlcA were observed in HAS2 KO cells, suggesting that these compounds may compensate for the loss of HA. Adding HA to the HAS2 KO cells reduced these expression levels (Fig. 4), further indicating that HA may regulate intracellular nucleotide sugars and their associated functions in cancer cells. These findings may represent an adaptive response in which cancer cells compensate for HA deficiency by upregulating UDP-GlcNAc–dependent glycosylation pathways. Importantly, this regulatory effect of HA on glycan metabolism and cellular behavior was not observed in CD44 KO cells, suggesting that HA-mediated responses require the presence of CD44. Consistently, CD44 KO also decreased β-catenin expression (76, 77), a key component of the Wnt pathway, which can be activated by HA binding to CD44.

Although the current study examined the effects of HA and CD44 on the β-catenin–GFAT axis only in HeLa and PANC-1 cells, which limits its diversity, other independent studies also support a functional connection among hyaluronan synthesis, GFAT, and β-catenin signaling across various tumor types. For example, studies in breast cancer have shown that increased flux through the HBP, along with higher levels of GFAT and HAS2, enhances Akt/GSK3β/β-catenin signaling and increases O-GlcNAcylation, promoting cancer stem–like properties (78). In pancreatic cancer, GFAT1-dependent O-GlcNAcylation helps maintain β-catenin activity and supports aggressive behavior (79). Similarly, in glioma models, HAS2 overexpression enhances malignancy and reduces ferroptosis *via* Wnt/β-catenin–dependent pathways (80). Therefore, it is necessary to expand the present analysis to other cancer models in the future.

Given the critical role of GFAT enzymes in hexosamine biosynthesis, we examined their expression after CD44 depletion. In HAS2 KO cells, *GFAT1* and *GFAT2* levels were elevated, paralleling changes in UDP-GlcNAc, whereas HA supplementation reversed these effects. These results suggest that the compensatory upregulation of UDP-GlcNAc and GFAT expression in HAS2 KO cells occurs only in the presence of CD44, which allows cells to sense residual HA and trigger feedback response. In contrast, CD44 KO cells exhibited decreased *GFAT1* and *GFAT2* expressions, along with a marked reduction in UDP-GlcNAc, supporting that the absence of CD44 interrupts HA sensing and downstream HBP activity, thereby preventing this feedback. Activation of the Wnt pathway by BIO treatment restored *GFAT1* and *GFAT2* expression as well as UDP-GlcNAc levels, supporting a model in which extracellular HA regulates intracellular glycosylation and malignant phenotypes *via* the CD44-β-catenin axis (Fig. 11). Thus, CD44 may function not only as a structural receptor for HA but also as a metabolic sensor, linking extracellular matrix composition to intracellular sugar nucleotide homeostasis. It is notable that additional pathways besides β-catenin may also contribute to positive or negative regulation. The detailed underlying mechanism requires further investigation. Other regulatory pathways may also modulate GFAT activation and UDP-GlcNAc levels under specific metabolic conditions. For example, under conditions of energy stress or metabolic insufficiency, such as nutrient deprivation or hypoxia, AMP-activated protein kinase (AMPK) can be activated as an energy sensor. Activated AMPK phosphorylates *GFAT1*, thereby reducing UDP-GlcNAc production and downstream protein glycosylation (81, 82). Given that HA depletion in our study was accompanied by increased *GFAT1* and *GFAT2* expression and enhanced glycosylation, it is possible that AMPK signaling is also involved in this process.

The functional effects of this regulatory pathway may be especially evident in cancer metastasis. Reductions in HA levels caused notable changes in glycosylation patterns, including increased expression of GlcNAc-branched *N*-glycans and *O*-GlcNAcylation, as well as UDP-GlcNAc and UDP-GlcA, as previously described, which may compensate for the loss of HA and reflect a feedback response to HA deficiency. The expression of GlcNAc-branched *N*-glycans and *O*-GlcNAcylation is closely associated with increased tumor cell mobility, invasiveness, and immune evasion (83, 84). Therefore, this study introduces a new regulatory mechanism for glycan biosynthesis involving HA, *N*-glycans, and *O*-GlcNAcylation, with UDP-GlcNAc serving as a common link. Furthermore, tumor cells detect extracellular HA in the tumor microenvironment through CD44 to regulate intracellular nucleotide sugar biosynthesis, affecting glycan metabolism. This process may influence tumor cell fate, influence the balance between proliferation and migration behaviors. It may also play a role in cancer metastasis processes involving EMT and mesenchymal–epithelial transition.

## Experimental procedures

### Antibodies and reagents

The experiments used the following antibodies and reagents: HA (18,237–41) from Nacalai Tesque; biotinylated E4-PHA (J211) and DSA (J205) from Mitsubishi Gas Chemical Company, Inc. (formerly JCM); the ABC kit (PK-4000) from Vector Laboratories; anti-O-GlcNAc (CTD110.6; 9875), OGA (60,406), CD44 (3570), β-catenin (8480), and P-β-catenin (9561) antibodies from Cell Signaling Technology; anti-α-Tubulin (T6199) and peroxidase-conjugated secondary antibody against mouse IgG (AP124P) from MilliporeSigma; goat anti-mouse IgG (Alexa Fluor 568) and streptavidin (Alexa Fluor 647 conjugate) were from Thermo Fisher Scientific; OGT antibody (ab177941) from Abcam; Ab-Capcher MAG beads from ProteNova; an Inertsil ODS-4 column (5020–14009) from GL Sciences; UDP-Glc.2Na (U4625–25 MG) and UDP-GlcNAc.2Na (U4375–25 MG) from MilliporeSigma; UDP-GlcUA.3Na (YM7095) from Yamasa; 2 mol/L triethylamine acetate, pH 7.0 (202–13131) from Wako; and Supelclean ENVI-Carb (57,088) from Supelco. Other solvents and reagents were of the highest grade commercially available.

### Cell lines and cell cultures

HeLa, PANC-1, HepG2, and MIA PaCa-2 cells were obtained from the RIKEN Cell Bank. A431 cells were obtained from ATCC. All cell lines were cultured in DMEM containing 10% fetal bovine serum (FBS) and incubated at 37 °C in a humidified environment with 5% CO_2_.

### Establishment of HAS2 KO and CD44 KO cells

The Lenti-CRISPR v2 plasmid (#52961) was obtained from Addgene (85). HAS2 KO cells were generated using a guide RNA (5′-CTCGCAACACGTAACGCAAT-3′) from the GeCKO library (86), cloned into the Lenti-CRISPR v2 vector to target the human HAS2 gene. Lentiviral particles were produced by co-transfecting 293T cells with the HAS2-targeting Lenti-CRISPR v2 together with psPAX2 (5.5 μg), pMD2.G (2.4 μg), and a small amount of TAX1 (0.6 μg) to enhance viral titers (87). Viral supernatants were collected 48 h after transfection and used to infect HeLa and PANC-1 cells. Cells were selected with puromycin (0.5 μg/ml), then seeded into 96-well plates for single-cell cloning. The target genomic region was PCR-amplified with primers F: 5′-CAGTTGCCCTTTGCATCG-3′ and R: 5′-TCTCAAGACCCTTCTGCTCAGG-3′, and sequencing confirmed homozygous disruption. These clones were designated as HAS2 KO cells.

The pSpCas9(BB)-2A-GFP plasmid (PX458; #48138) was obtained from Addgene (88). CD44 KO cells were generated using a single-guide RNA (5′-CCGTCCGAGAGATGCTGTAG-3′) targeting the human CD44 gene, cloned into the pSpCas9(BB)-2A-GFP vector adjacent to the Cas9 coding sequence. The stable CD44 KO cell line was established by electroporating HeLa cells according to the manufacturer’s protocol (Amaxa Nucleofector Kit; Lonza). At 24 h post-transfection, GFP-positive cells were isolated with a FACSAria fusion cell sorter (BD Biosciences), serially diluted, and seeded into 96-well plates for single-cell cloning. Individual clones were expanded over a period of 4 weeks. Genomic DNA from expanded clones was PCR-amplified with primers F: 5′- ACCTGCCGCTTTGCAGGTGTA-3′ and R: 5′- ATGGCCCAGATGGAGAAAGC -3′, and sequencing confirmed a homozygous frameshift mutation in CD44. These clones were designated as CD44 KO cells.

### Establishment of rescue and overexpression cell lines

The methods for viral production and infection followed previous protocols (89, 90). To restore HAS2 or overexpress CD44 in HAS2 KO cells, lentiviral vector pLX303-HAS2-3 × FLAG (cloned into the pLX303 backbone; a gift from David Root, Addgene plasmid #25897) (91) and the retroviral vector pWZL-Blast-CD44S (a gift from Bob Weinberg, Addgene plasmid #19126; RRID: Addgene_19126) (92) were independently packaged into viral particles. HAS2 KO HeLa cells were infected with the respective viral supernatants in the presence of 2 μg/ml polybrene (Millipore Sigma). After 48 h, cells were selected with 2 μg/ml blasticidin for 5 days. Stable cell lines were expanded and validated *via* Western blot. The resulting lines were named HAS2 KO RES (pLX303-HAS2-3 × FLAG) and HAS2 KO + CD44 OE (pWZL-CD44S).

### Immunoprecipitation

Cells were washed twice with ice-cold PBS to remove any residual medium. Then, cells were lysed on ice for 30 min using lysis buffer (20 mM Tris, pH 7.4, 150 mM NaCl, 1% Triton X-100) containing protease and phosphatase inhibitors (Nacalai Tesque). The lysates were centrifuged at 16,000*g* for 15 min at 4 °C. The supernatant was collected, and the pellet discarded. Protein concentration was measured using a BCA Protein Assay Kit (Wako) according to the manufacturer’s instructions, with bovine serum albumin (BSA) as the standard.

For immunoprecipitation, 1.5 μl of anti-CD44 antibody was mixed with 15 μl of Ab-Capcher MAG at 4 °C for 2 h using the MT 360 Micro Tube Mixer. After washing the mixture three times with TBS (20 mM Tris, pH 7.4, 150 mM NaCl), the beads were incubated with 500 μg of cell lysate overnight at 4 °C. The immunoprecipitates were then washed twice with TBS and analyzed by Western blot.

### Western blot and lectin blot

Western blotting and lectin blotting were performed essentially as described previously (90), with minor modifications as detailed below.

Equal amounts of protein (10 μg) were resolved on 7.5% SDS–PAGE at 100 V and transferred to PVDF membranes at 10 V for 1 h. Membranes were blocked with 5% non-fat milk or BSA for 1.5 h at room temperature, then incubated overnight with primary antibodies at 4 °C. After three 10-min washes with TBST, secondary antibodies were applied for 1 h at room temperature. According to the manufacturer’s instructions, immunoreactive bands were detected using an Immobilon Western Chemiluminescent HRP Substrate (MilliporeSigma).

### Flow cytometry analysis

Cells (1 × 10^6^) were washed twice with PBS and incubated with biotinylated lectin (E4-PHA and DSA) diluted in cell sorting buffer (PBS + 0.1% BSA) on ice for 30 min with gentle mixing every 10 min. After washing, cells were stained with streptavidin-conjugated Alexa Fluor 647 (1:3000 dilution) in the dark on ice for 30 min. Cells were washed twice and resuspended in 800 μl of cell sorting buffer. Fluorescence intensities were detected using the Attune N x T Flow Cytometer (Thermo Fisher Scientific) according to standard flow cytometry protocols (93) and analyzed with FlowJo v10 (BD Biosciences).

### RNA extraction and quantitative real-time PCR (qPCR) for the detection of mRNA expression levels

Total RNA was extracted using TRI Reagent (MilliporeSigma), and 1 μg of total RNA was reverse-transcribed into complementary DNA with PrimeScript RT reagent containing gDNA Eraser (Takara Bio) following the manufacturer’s instructions. The specific primer sequences are listed in Table 1. PCR products were diluted to 50 ng/μl and quantified using a StepOnePlus Real-Time PCR System (Applied Biosystems). Amplifications were carried out with TB Green Premix Ex Taq II (Tli RNaseH Plus) (Takara Bio) under the following cycling protocol: an initial denaturation at 95 °C for 30 s; then 40 cycles of denaturation at 95 °C for 5 s, and combined annealing and extension at 60 °C for 30 s.

**Table 1:** Primer sequences for qPCR

Gene names	Forward primer	Reverse primer
HAS1	TCAAGGCGCTCGGAGATTC	CTACCCAGTATCGCAGGCT
HAS2	ATGCATTGTGAGAGGTTTC	TCATACATCAAGCACCATG
HAS3	CGCAGCAACTTCCATGAGG	AGTCGCACACCTGGATGTAGT
MGAT1	TGACCAGCACCTCAAGTTTATC	CGGAACTGGAAGGTGACAATAC
MGAT2	AGAGTGCCCTGAATGTGATG	CACAGTCTCCAGCATGAAAGA
MGAT3	GCCGCGTCATCAACGCCATCAA	CAGGTAGTCGTCGGCGATCCA
MGAT4A	GGCTATCACACCGATAGCTGGAG	TCCACCATTCCTTCTGCAACACC
MGAT4B	ACAACCCTCAGTCAGACAAGGAGG	GGTACCCTCAGAAGCCCGCAGCTT
MGAT5	GACCTGCAGTTCCTTCTTCG	CCATGGCAGAAGTCCTGTTT
OGT	GTTCTTGCTGGTAGTGGCGG	ACGTTTCGTTGGTTCTGTGCT
OGA	GAAGGAGAGTCAAGCGACGTT	TCCATAACCCAAGGTCTTCCAT
GFAT1	GCGGCCTGTGGTAATTTGTG	CAAGGTGGAAAGCCAGCAAC
GFAT2	TTGGTCGAGAGAGTCATTCAGC	AAGATAGGGATCTGTTCTGTGGA
GAPDH	ACTCCACTCACGGCAAATTC	CCCTGTTGCTGTAGCCGTAT

### Quantification of hyaluronic acid synthesis in HeLa WT and HAS2 KO cells

HeLa WT and HAS2 KO cells were seeded at 4 × 10^5^ cells per well in 6-well plates and cultured under standard conditions. After 48 h, the medium was replaced with serum-free medium to eliminate exogenous HA; conditioned medium was collected 24 h later. HA concentration was quantified using the HTRF Hyaluronic Acid Assay Kit (Cisbio, 63ADK089PEG, Lot 12) according to the manufacturer’s protocol.

### HPLC

Nucleotide sugars were purified from cells following the previously described method with minor modifications (94, 95). HeLa cells were cultured in 10 cm dishes with DMEM containing 10% FBS for 2 days until reaching 70 to 80% confluence. The cells were washed twice with PBS and collected by scraping into 70% ethanol and then sonicated and centrifuged at 16,000*g* for 10 min at 4 °C to collect the supernatant. The supernatant was frozen and lyophilized. The lyophilized samples were dissolved in 10 mM ammonium bicarbonate (1 ml) and subjected to solid-phase extraction using an ENVI-Carb column (250 mg). The column was conditioned with 80% acetonitrile in 0.1% trifluoroacetic acid (2 ml) and washed with Milli-Q water (1 ml). After loading the sample, the column was washed sequentially with the following solutions: Milli-Q water (1 ml), 25% acetonitrile (1 ml), Milli-Q water (2 ml), and 50 mM triethylamine acetate buffer (pH 7, 1 ml). Nucleotide sugars were eluted with 25% acetonitrile in 50 mM triethylamine acetate buffer (pH 7, 1 ml), and the eluate was lyophilized again. Quantification of nucleotide sugars was performed by ion-pair reversed-phase HPLC with detection at 254 nm. Separation was achieved using an ODS-4 column (4.6 × 250 mm) equipped with a guard column and maintained at 40 °C. The mobile phases consisted of Buffer A (200 mM triethylamine acetate, pH 6.0) and Buffer B (70% Buffer A + 30% acetonitrile). Lyophilized samples were dissolved in 100 μl of Milli-Q water, and 10 μl was injected. The gradient program consisted of the following steps: 100% Buffer A for 35 min, 0 to 77% Buffer B over 40 min, 77 to 100% Buffer B over 1 min, and 100% Buffer B for 14 min. Chromatographic peaks were integrated and quantified using an external standard curve, and absolute amounts were normalized to cell number and reported as pmol per 10^6^ cells.

### Transwell assay

Transwell migration assays were performed according to standard methods (96). Transwell inserts were coated overnight at 4 °C with 10 μg/ml fibronectin, followed by blocking with 1% BSA in PBS at 37 °C for 1 h. Cells (1 x 10^5^), suspended in 400 μl of serum-free medium, were seeded into the upper chamber. The lower chambers of 24-well plates were filled with DMEM containing 10% FBS or the same medium supplemented with HA (200 ng/ml). After 16 h of incubation, cells were fixed with methanol and stained with 0.1% crystal violet solution for 25 min. Migrated cells were counted in three randomly selected fields per chamber under a light microscope.

### Wound-healing assay

Wound-healing assays were performed as described previously, with minor adaptations (97). Cells were seeded at a density of 1 × 10^6^ cells per well in 6-well plates and allowed to grow until a confluent monolayer formed. A uniform scratch was created in the monolayer using a sterile 200 μl pipette tip. Cells were then incubated for 16 h in DMEM supplemented with 10% FBS or the same medium containing HA (200 ng/ml). After incubation, the medium was removed, and non-adherent cells were washed away with PBS. Wound areas were imaged at 0 h and 16 h using a phase-contrast microscope (Olympus, IX71). Cell migration was quantified by measuring the distance between the wound edges using ImageJ software 1.5.

### Colony assay

Cells (1 × 10^3^) were seeded into 3.5 cm dishes and cultured in medium supplemented with 10% FBS at 37 °C. The medium was changed twice a week. After 2 weeks, the cells were fixed with methanol for 20 min and stained with 0.1% crystal violet for 25 min. The stained colonies (>50 cells per colony) were manually counted to assess colony-forming capacity.

### Cell proliferation assay

Cell proliferation was measured by direct cell counting. Initially, 5 × 10^3^ cells were seeded into 48-well plates and cultured in DMEM with 10% FBS at 37 °C. Cell numbers were recorded every 24 h for up to 4 days. Growth curves were generated as cell count *versus* time.

### Statistical analysis

All data are presented as the mean ± SD from at least three independent experiments. Statistical analyses were conducted using a one-way ANOVA with Tukey’s *post hoc* test, a two-way ANOVA with Tukey’s multiple comparisons test, or an unpaired Student's *t* test, all performed with GraphPad Prism 6.0 software (GraphPad Software, Inc.). A *p* value was considered significant as follows: ns (no significance) for *p* > 0.05; ∗*p* < 0.05; ∗∗*p* < 0.01; and ∗∗∗*p* < 0.001.

## Data availability

All data presented in the figures and tables of this paper are available.

## Supporting information

This article contains supporting information.

## Conflicts of interest

The authors declare no conflicts of interest and no competing financial interests.
