# Supplementary material for: Hyaluronic acid regulates cellular UDP-GlcNAc levels through CD44 to affect glycosylation and cell biological functions
Source: J Biol Chem. 2025 Dec 27;302(2):111111. doi: 10.1016/j.jbc.2025.111111 (PMC12830202; doi:10.1016/j.jbc.2025.111111)
Supplement: Supporting information [file mmc1.pdf]

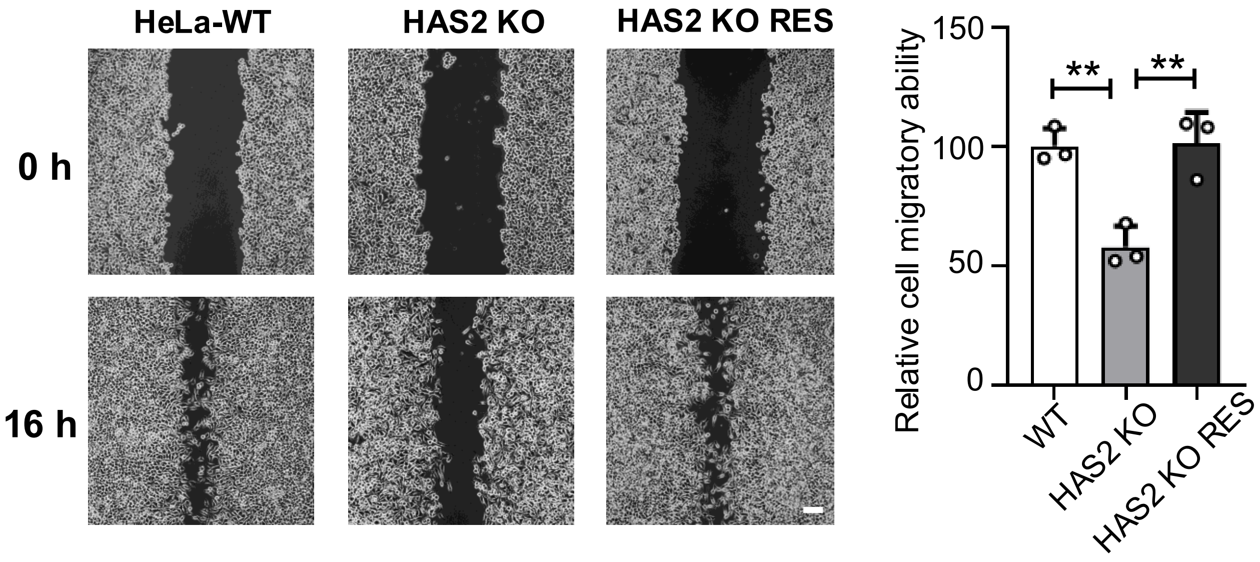

sFig.1. Effects of restoration with the HAS2 gene on cell migratory capacity in HAS2 KO cells. WT, HAS2 KO, and HAS2 KO RES cells restored by reintroducing *HAS2* into the HAS2 KO cells were cultured for 16 h following scratching in the wound-healing assay. Representative images of WT and HAS2 KO cells, shown in Figure 10C, and the HAS2 KO RES cells were obtained from the same experimental batch of a wound-healing assay performed in three independent experiments. The migration ability of WT cells was set at 100%. All values are expressed as mean ± SD, analyzed with one-way ANOVA and Tukey's post hoc test. $**p < 0.01$.

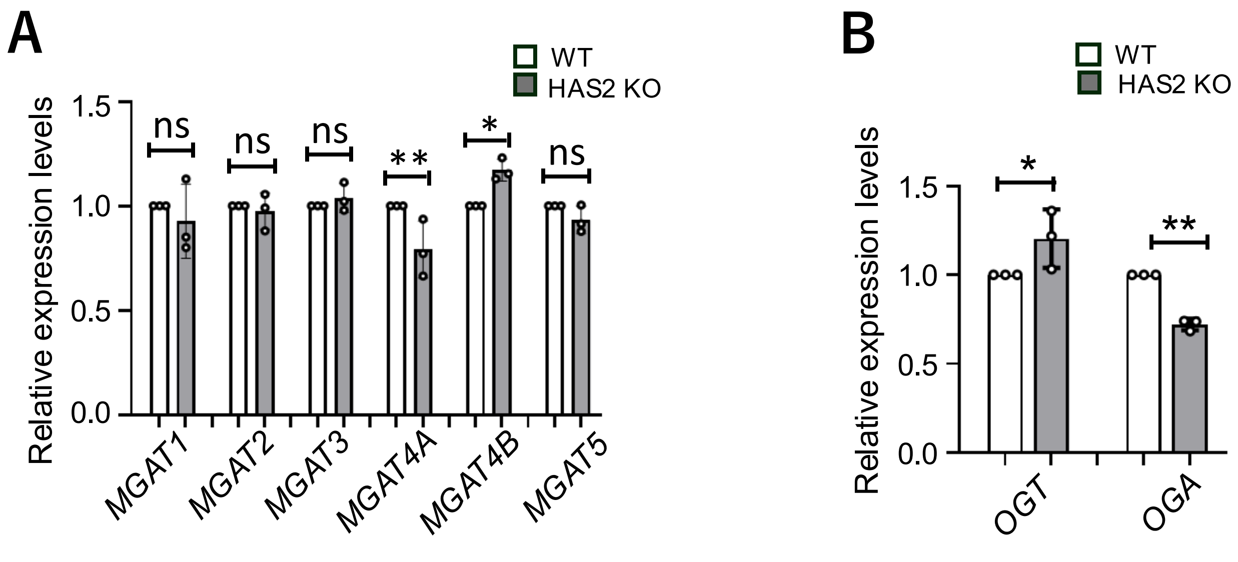

sFig.2. Effects of HAS2 KO on the expression of some glycogenes related to *N*-glycan branching and *O*-GlcNAcylation.

A, mRNA levels of several *N*-acetylglucosaminyltransferases involved in synthesizing GlcNAc-branched *N*-glycans were determined using qPCR in WT and HAS2 KO cells. *GAPDH* was used as an internal control, and values were normalized to WT cells (set as 1.0). Statistical significance was assessed from three independent experiments using the unpaired Student's t test, with *p*-values indicated as **$p < 0.01$, *$p < 0.05$, or no significance (ns).

B, mRNA levels of *OGT* and *OGA* were determined using qPCR in WT and HAS2 KO cells. *GAPDH* was used as an internal control, and values were normalized to WT cells (set as 1.0). Statistical significance was assessed from three independent experiments using an unpaired Student's t-test, with *p*-values indicated as **$p < 0.01$, *$p < 0.05$.

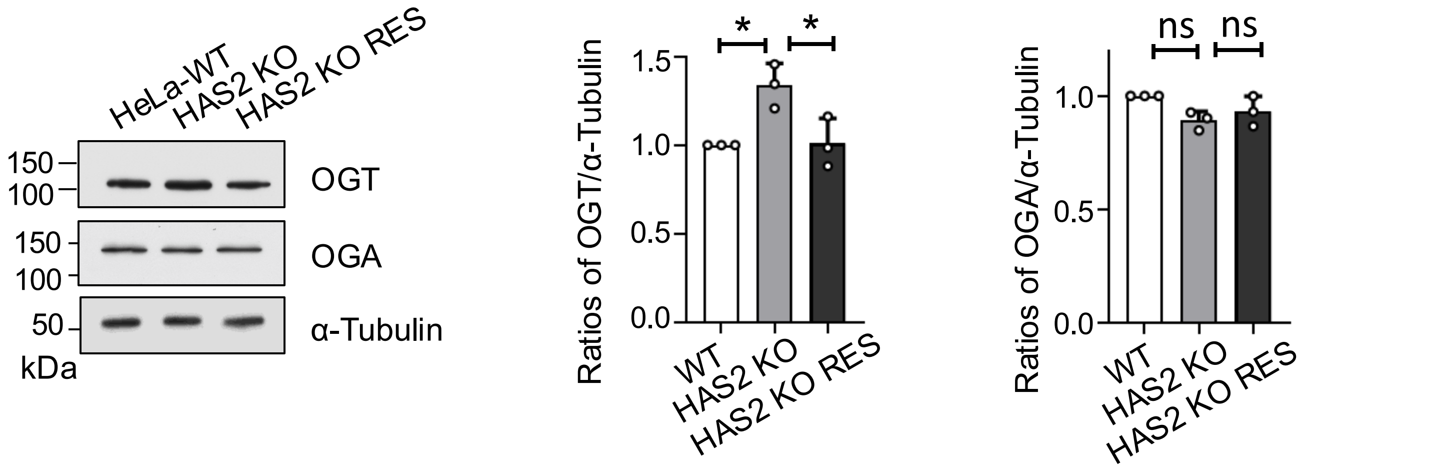

sFig.3. Effects of restoration with the HAS2 gene on OGT and OGA expression in HAS2 KO cells.

Equal amounts of cell lysate from WT, HAS2 KO, and HAS2 KO RES cells were analyzed by Western blot with OGT and OGA antibody. α-Tubulin served as a loading control. The ratios of OGT to α-Tubulin and OGA to α-Tubulin in WT cells were set as 1.0. Data are shown as mean ± SD from three independent experiments. *p*-values were calculated by one-way ANOVA with Tukey's post hoc test. *$p < 0.05$ or no significance (ns).
